# Diffusion-Weighted Magnetic Resonance Imaging of an Intramedullary Epidermoid Cyst with Dorsal Dermal Sinus Tract in a Toddler

**DOI:** 10.1155/2013/878713

**Published:** 2013-10-22

**Authors:** Michael G. Fazio, Alyson M. Kil, Veronica J. Rooks, Timothy J. Biega

**Affiliations:** ^1^Department of Radiology, MCHK-DR, Tripler Army Medical Center, 1 Jarrett White Road, Honolulu, HI 96859, USA; ^2^Keck School of Medicine, University of Southern California, Los Angeles, CA, USA

## Abstract

We report the use of diffusion-weighted magnetic resonance imaging to diagnose and manage a rare case of a symptomatic thoracic intramedullary congenital epidermoid cyst with associated dermal sinus in a girl. Congenital intramedullary epidermoid cysts with associated dermal sinuses are very rare occurrences and seldom present symptomatically in very young children. We present a case of a 32-month old with a draining dimpled skin lesion. Magnetic resonance images demonstrated an intramedullary epidermoid with a dorsal dermal sinus tract opening to the skin surface which was confirmed surgically. The patient was treated with debulking to prevent recurrent infection and progression of neurological symptoms. This case demonstrates the use of diffuse-weighted MRI to assist in the diagnosis and surgical management of an atypical presentation of a rare developmental abnormality, which is not well documented in the pediatric radiological literature. Failure to diagnosis may have significant neurological permanent debilitating consequences.

## 1. Introduction

Dermal sinuses are rare abnormalities which result from abnormal cleavage of cutaneous ectoderm from the neuroectoderm. This creates an elongated dermal tube that extends interiorly from the surface and creates a communication from the central nervous system to the skin [1]. The sinus tract may expand to form an epidermoid tumor, a cystic lesion lined by squamous epithelium, which may necessitate surgical removal [2]. Together, spinal epidermoids and dermoids account for approximately 1% of primary spinal tumors. They are more frequent in males. Approximately 60% of epidermoids are intradural extramedullary and 40% are intramedullary. In approximately 5% of cases, there are multiple lesions. An infected dermal sinus may cause abscess formation, or extension from the medulla to the conus medullaris, or a meningeal infection. We report a case of a toddler with an intramedullary epidermoid and dorsal dermal sinus diagnosed with magnetic resonance imaging. The clinical course, pathogenesis, imaging characteristics, and management are discussed. 

## 2. A Case Report

A 32-month-old female presented to the emergency department with recurrent upper respiratory infections and fever. History revealed that the patient was born with a dimpled skin lesion on her upper back that was noted to occasionally drain fluid. She was neurologically intact. Physical exam-ination demonstrated several draining pustules on her buttocks. The patient was diagnosed with folliculitis and scheduled for followup with her primary care physician. On follow-up visit, the patient was noted to have a nonfluctuant, warm 2 mm posterior mid-thoracic red papule which was diagnosed as a furuncle. A dimpled skin lesion on the patient's upper back was also noted, and the toddler was referred to pediatrics. The pediatrician described the appearance of a sinus tract on the upper back with some crusted drainage from the center without surrounding erythema or tenderness. 

Following the pediatrician visit, the lesion demonstrated increased erythema, tenderness, and occasional drainage. The patient began to walk with her back hunched to alleviate some of the pain. No weakness or neurological deficits was present. On subsequent physical examination, after approximately 2 weeks, the lesion had grown to become a 2 cm flesh-colored mass with a central dimple and a crusted papule. A diagnosis of a thoracic dermal sinus tract with a possible underlying mass lesion was made. The draining fluid was cultured and the patient was treated empirically with clindamycin. 

**Figure 1:**
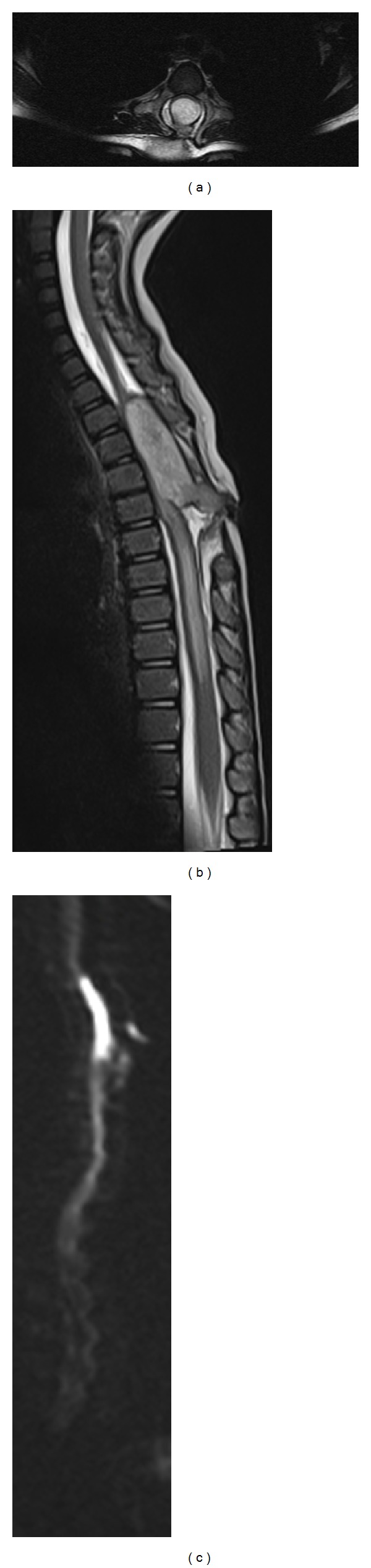
Axial T2-W (a), sagittal T2-W (b), and DW (c) MR images. (a) A hyperintense well-defined, round, intramedullary lesion is seen spanning T3–T6 vertebral levels. (b) The hyperintense lesion demonstrates continuity with the skin surface consistent with a dorsal sinus tract. (c) There is hyperintensity of the spinal lesion on the sagittal DW image consistent with restricted water diffusion.

**Figure 2:**
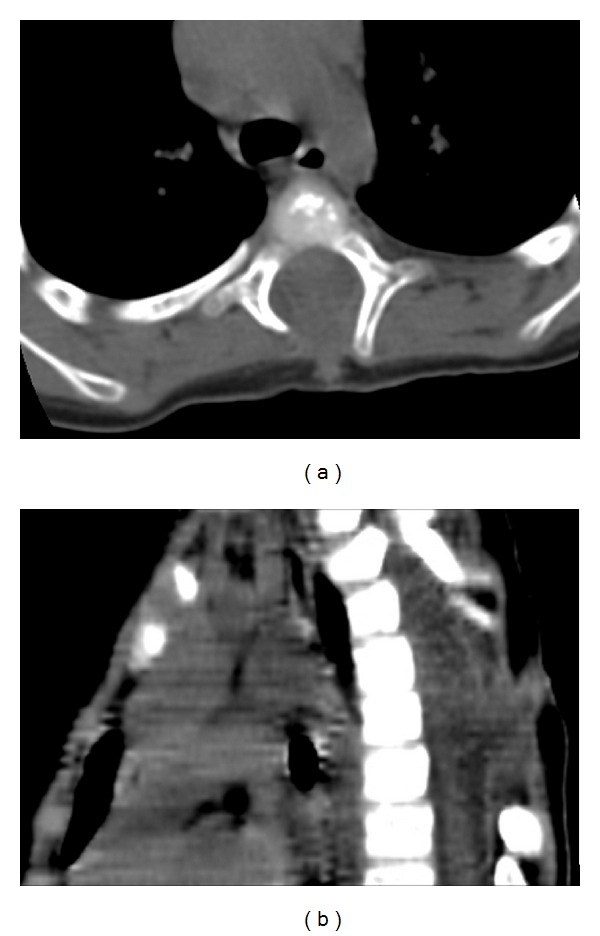
Axial (a) and sagittal (b) CT images. There is deficiency of the posterior elements at T3–T6, with remodeling and widening of the spinal canal. A soft tissue attenuation tract extends to the skin surface.

Thoracic spine radiographs demonstrated widening of the interpediculate distance from T3 through T8 and ill-defined posterior portions of the vertebral bodies with concern for intraspinal mass or congenital abnormality. CT of the spine demonstrated herniation of the thecal sac posteriorly, consistent with a meningocele, and soft tissue attenuation at the skin surface, consistent with the dimple seen on physical examination. Defects within the posterior aspects of the T3 through T6 vertebral bodies were seen (Figure 1). Subsequently, MR imaging including diffuse-weighted images sequences was performed demonstrating a nonenhancing intramedullary lesion hypointense on T1-weighted images and hyperintense on T2-weighted images with restricted diffusion in the thoracic region, consistent with an epidermoid (Figure 2). The intramedullary lesion was contiguous with a tract which opened to the skin surface, consistent with a dermal sinus. 

The patient, now 34 months old, had developed an abnormal wide-based gait and was referred to neurosurgery. A thoracic laminectomy was performed from T2 to T7 with resection of the intramedullary epidermoid and subfascial dermal sinus tract. Ten months later, follow-up MRI was performed which demonstrated small residual epidermoid cyst at the levels of T3 and T4. Two years after initial operation, patient underwent debulking and partial resection of a recurrent thoracic midline epidermal cyst. Follow-up examinations revealed normal neurologic function, and imaging studies showed expected postoperative findings without appreciable remaining enhancing mass. 

## 3. Discussion 

There is a diverse group of cystic lesions of the spine which may be grouped by etiology (infectious, iatrogenic, posttraumatic, or degenerative abnormalities) or more commonly by location. MRI can be useful in diagnosis, by identifying the size of the cyst component, multiplicity, the compartment in which they lie, and involvement into adjacent compartments. Perhaps most importantly, MRI in conjunction with CT may characterize the tissue type of the intracystic contents.

Congenital epidermoid cysts grow slowly and therefore often present after early childhood. Acquired epidermoids may occur secondary to lumbar puncture, surgery, or trauma. Presentation may range from back pain, radiculopathy, myelopathy, infection, or symptoms of chemical meningitis from rupture. 

MR imaging demonstrates a mass hypointense or isointense on T1-W and hyperintense on T2-W images, often similar to CSF. Diffusion restriction provides a definitive diagnosis of an epidermoid cyst, distinguishing the cystic structure from arachnoid cysts which show no restriction of diffusion. On CT, epidermoids have low density very similar to CSF. There may be remodeling and widening of the spinal canal as seen in our case.

Dorsal dermal sinuses are rare congenital abnormalities. Those are caused by an abnormal cleavage of the cutaneous ectoderm from the neuroectoderm between the third and fifth weeks of gestation [3, 4]. Any variation in this complex process could result in abnormal accumulation of cutaneous components, leading to the formation of a dermoid or epidermoid cyst [5]. The formation of a sinus tract could occur at any point along the closure of the neural tube during neurulation, but more frequently occurs at the lumbosacral or suboccipital region due to the caudal and cranial migration of the neural tube closure. 

Our patient was diagnosed after MR imaging demonstrated a large cystic structure consistent with an epidermoid cyst with a dorsal dermal sinus tract. Our case demonstrates an unusual presentation of an epidermoid cyst, in which our patient was female, became symptomatic at a very young age, occurred in the thoracic region, and had an associated dorsal dermal sinus tract, which is more frequently seen with dermoid cysts. Although there are case reports of epidermoid cysts and dermal sinus tracts, to our knowledge, this is the first case report of diffusion-weighted imaging utilized to preoperatively confirm the epidermoid.

Definitive management of epidermoid and dermoid cysts is complete removal of the cyst capsule. Additionally, the dorsal dermal sinus should be surgically excised in order to prevent recurrence. Urgent operation is indicated in those with rapid onset of neurological deficit or signs of central nervous system infection in order to prevent permanent neurologic damage or spread of infection. Despite aggressive surgical management, in many cases the cystic structures recur, in which case reexploration is performed. 

To conclude, we present a rare presentation of an uncommon entity, for which MR imaging with DWI plays an important role in preoperative diagnosis and surgical planning. Radiologists should be aware of the necessity to perform DWI on intraspinal cystic lesions to aid in the diagnosis of epidermoid cyst.
